# DMAPLM: A multimodal pretrained framework for computational drug repositioning

**DOI:** 10.1371/journal.pcbi.1014192

**Published:** 2026-04-22

**Authors:** Hailin Chen, Zhongling Li

**Affiliations:** School of Information and Software Engineering, East China Jiaotong University, Nanchang, China; Washington University in Saint Louis, UNITED STATES OF AMERICA

## Abstract

Drug repositioning offers an efficient route to discover new therapeutic indications for existing drugs. However, current computational drug repositioning models often face challenges related to data scarcity, heterogeneity, and therefore limited generalizability. To address these limitations, this study introduces DMAPLM, a multimodal pretrained framework for predicting drug-disease associations for further drug repositioning screening. DMAPLM leverages a lightweight dual-encoder architecture, utilizing ChemBERTa-2 for molecular encoding of drug SMILES strings and BioBERT for semantic encoding of multi-field disease texts. The framework explicitly aligns drug and disease representations through contrastive learning and employs attention-weighted pooling to emphasize informative molecular substructures. A Random Forest classifier is finally used for association prediction based on the enhanced multimodal features. We compile a new and comprehensive benchmark dataset for performance evaluation. Extensive experiments demonstrate that DMAPLM significantly outperforms six state-of-the-art baseline models, achieving an AUROC of 0.8919 and AUPR of 0.9116 under five-fold cross-validation, representing an improvement of up to 9%. Furthermore, DMAPLM exhibits robust performance in challenging cold-start scenarios, highlighting its practical utility for identifying novel drug-disease relationships. Case studies along with molecular docking analysis confirm the interpretability and biological meaningfulness of our predictions. Our study provides a powerful and interpretable approach for computational drug repositioning.

## Author summary

Identifying potential associations between drugs and diseases is essential for accelerating drug discovery and repurposing, yet current computational approaches often struggle when data are sparse or when new drugs or diseases lack sufficient annotations. To address these challenges, we propose a cross-modal learning framework that jointly models molecular structures and disease text descriptions. By explicitly aligning these two information sources through contrastive learning and attention mechanisms, our method captures complementary patterns that conventional models tend to overlook. As a result, the framework provides robust predictions even in cold-start scenarios and offers biologically interpretable insights that can support real-world translational applications. Our study demonstrates the promise of integrating chemical and biomedical language representations to better understand drug–disease relationships and guide future drug development.

## Introduction

The pharmaceutical industry is continually confronted with significant challenges in the process of drug development, including escalating research and development costs, prolonged timelines, and high failure rates [1]. In response, drug repositioning [2]-also known as drug repurposing-has garnered considerable attention as an efficient strategy to identify novel therapeutic indications for existing drugs. By leveraging approved drugs or those in clinical development for new usage, this approach offers a markedly reduced risk profile relative to *de novo* drug discovery, largely owing to the established safety, pharmacokinetic, and pharmacodynamic profiles of the existing drugs [3]. Consequently, drug repositioning holds immense potential to expedite the availability of treatments for unmet medical needs, particularly for rare diseases and emergent health threats, yielding notable clinical and economic benefits.

Although traditional experimental drug repurposing, initiated by hypotheses on a drug’s mechanism of action or *in vitro* screening of cell lines and animal models against specific disease targets, has yielded successes, it is frequently hampered by low efficiency, limited scope, and a high degree of unpredictability in identifying a broad spectrum of clinically viable repurposed drug candidates [4,5]. Moreover, existing experimental approaches are often biased by prior knowledge, potentially overlooking unconventional yet effective drug-disease associations.

With the accumulation of various types of biomedical data, such as chemical structures, gene expression profiles and adverse event profiles, researchers have developed a series of computational methods to predict novel drug-disease associations for further drug repositioning screening. These methods can be roughly divided into three categories, namely network propagation-based [6–8], traditional machine learning-based [9–11] and deep learning-based [12–14]. More recently, with the emergence of large language models (LLMs) such as ChatGPT, biomedical AI has entered a new era. Pretrained models, such as BioBERT [15], ChemBERTa [16], and ESM-2 [17], have shown strong feature representation capabilities by learning from large-scale corpora, and successfully applied to tasks such as molecular property prediction [18] and protein design [19]. Some recent studies have applied LLMs to drug repurposing [20–23].

Despite these impressive progress, current computational drug repurposing methods face the following challenges and limitations. Firstly, current gold-standard datasets, such as the well-known Gottlieb dataset [24], for training and evaluating drug repositioning prediction models are small and outdated, and are therefore missing many associations. Secondly, data scarcity and heterogeneity remain a persistent problem. While many drug and disease databases exist, they are often incomplete, biased, and lack standardization, hindering the robustness and generalizability of prediction models. Thirdly, the interpretability and explainability of complex computational models, particularly deep learning architectures, are often lacking. This “black box” nature can limit trust and hinder the translation of predictions into actionable insights for experimental validation. Finally, the integration of multi-modal data (e.g., genomics, proteomics, transcriptomics, electronic health records) to create comprehensive prediction models is still in its initial phases, and effectively analyzing such disparate data sources presents considerable technical challenges.

To address these problems, we compile a new benchmark dataset by searching molecular atlas and pharma-information from DrugMAP 2.0 (released in 2024) [25], and propose DMAPLM, a lightweight dual-encoder framework based on pretrained language models (PLMs), for drug-disease association prediction in this study. Unlike existing methods, DMAPLM directly integrates embeddings of drug molecules and disease texts learned from pretrained language models through contrastive learning and attention mechanisms, without requiring graph construction or task-specific fine-tuning, making it especially suitable for data-sparse and cold-start scenarios.

We comprehensively evaluate the performance of DMAPLM on the collected benchmark dataset. Experimental results show that under five-fold cross-validation and cold-start settings, DMAPLM improves AUROC and AUPR by up to 9% over state-of-the-art methods. Case studies along with molecular docking analyses further confirm that predicted associations align with literature-supported biological mechanisms, highlighting DMAPLM’s potential for real-world drug repurposing applications. The main contributions of this study are as follows:

1. Lightweight cross-modal architecture: We propose a dual-encoder framework that leverages pretrained language models-specifically ChemBERTa and BioBERT-to extract molecular and textual representations without constructing complex graph structures.
2. Explicit cross-modal alignment: We apply contrastive learning to explicitly align molecular structure and disease text semantic spaces, while capturing complementary features through attention mechanisms to enhance cross-modal discriminability.
3. Robustness and interpretability: The framework achieves stable performance under data-sparse and cold-start scenarios, with biologically interpretable predictions suitable for real-world clinical translational applications.

This paper is organized as follows. Section II presents the datasets and our proposed method, section III reports experimental results, section IV discusses the findings, implications, and limitations, and section V concludes the study.

## Materials and methods

### A. Datasets

To obtain the latest information for performance evaluation, we search the DrugMAP 2.0 (https://idrblab.org/drugmap) [25], a manually curated pharmaceutical database, for experimentally confirmed drug-disease associations. We retain only associations with “Approved” status to focus on clinically validated drugs. Meanwhile, we only keep drugs with valid SMILES (Simplified Molecular-Input Line-Entry System) representations. For diseases, we extract three of their textual fields from the DrugMAP 2.0 database: Disease Name, Synonymous, and Definition.

After data preprocessing, we finally construct a benchmark dataset comprising 1,455 diseases, 2,622 drugs, and 5,993 drug-disease associations, with an association density of 0.0016 (0.16%).

### B. Method architecture

**Fig 1:**
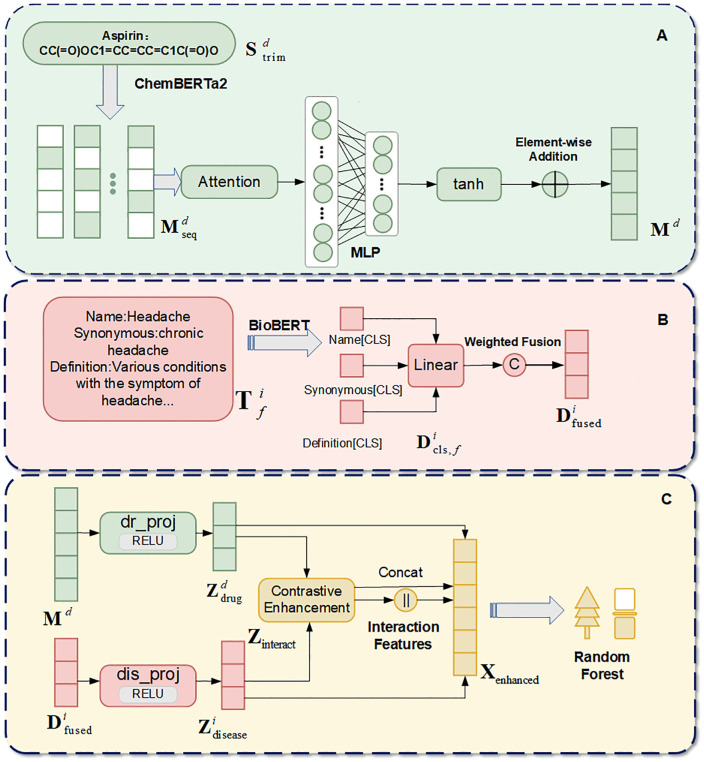
Workflow of DMAPLM for predicting drug-disease associations. Module A: ChemBERTa2 encodes SMILES sequences into 384-dimensional embeddings via attention pooling. Module B: BioBERT encodes multi-field disease texts with weighted fusion. Module C: Embeddings enhanced by contrastive learning for Random Forest prediction.

The model architecture of DMAPLM for drug-disease association prediction is illustrated in Fig 1, which mainly consists of three components: (A) attention-based molecular encoding using ChemBERTa-2 [26], (B) multi-field disease text encoding using BioBERT [15], and (C) contrastive learning enhancement for feature extraction with Random Forest-based classification.

#### Pre-trained language module for molecular and biomedical text encoding.

For drug molecules, we employ ChemBERTa-2 (DeepChem/ChemBERTa-77M-MTR) as the molecular encoder to transform drug SMILES strings into contextualized embeddings. ChemBERTa-2 is a RoBERTa-like pre-trained language model specifically designed for molecular representation learning based on SMILES notation. The model was trained on 77 million drug molecules from PubChem through self-supervised learning, enabling it to capture the intrinsic chemical semantics and structural patterns of molecular compounds. For each drug molecule, the SMILES string is first tokenized using the ChemBERTa-2 tokenizer, with sequences truncated to a maximum length of 512 tokens to ensure computational efficiency. The molecular encoding process is formulated as:


$$\overset{d}{\underset{\text{trim}}{𝐒}}=\text{Tokenizer}\left({\text{SMILES}}^{d}\right),$$ (1)

$$\overset{d}{\underset{\text{seq}}{𝐌}}=\text{ChemBERTa2}\left(\overset{d}{\underset{\text{trim}}{𝐒}}\right),$$ (2)

where $\overset{d}{\underset{\text{trim}}{𝐒}}\in {\mathbb{R}}^{L_{d}}$ represents the tokenized and truncated SMILES sequence for drug d with length $L_{d}\leq 512$, and $\overset{d}{\underset{\text{seq}}{𝐌}}\in {\mathbb{R}}^{L_{d}\times 384}$ denotes the contextualized molecular embeddings extracted from the final hidden layer of ChemBERTa-2. Each token is encoded as a 384-dimensional vector, capturing the sequential dependencies and local chemical environments within the molecular structure. These token-level representations preserve fine-grained structural information that will be subsequently aggregated to obtain drug-level representations for downstream prediction tasks.

For disease entities, we apply BioBERT (dmis-lab/biobert-base-cased-v1.1) as the disease text encoder to transform disease textual descriptions into semantic embeddings. BioBERT is a domain-specific pre-trained language model adapted from BERT (Bidirectional Encoder Representations from Transformers) for biomedical text mining. The model was pre-trained on large-scale biomedical corpora including PubMed abstracts and PMC full-text articles, enabling it to effectively capture complex biomedical terminologies and semantic relationships in disease descriptions. To construct comprehensive disease representations, we extract three complementary textual fields for each disease: Disease Name, Synonymous terms, and detailed Definitions. These multi-field descriptions are separately encoded and fused through weighted aggregation. For each textual field f, the encoding process is formulated as:


$$\overset{i}{\underset{f}{𝐓}}=\text{Tokenizer}\left(\overset{i}{\underset{f}{\text{Text}}}\right),$$ (3)

$$\overset{i}{\underset{\text{cls},f}{𝐃}}=\text{BioBERT}(\overset{i}{\underset{f}{𝐓}}{)}_{\left[\text{CLS}\right]},$$ (4)

where$\overset{i}{\underset{f}{𝐓}}$ represents the tokenized text sequence for disease i and fieldf truncated to a maximum length of 512 tokens, and $\overset{i}{\underset{\text{cls},f}{𝐃}}\in {\mathbb{R}}^{768}$ denotes the disease representation extracted from the [CLS] token [27,28] of BioBERT’s final hidden layer. The [CLS] token serves as an aggregate representation of the entire input sequence, capturing the global semantic meaning of the disease description. To leverage information from multiple textual fields, we perform weighted fusion across different fields as follows:


$$\overset{i}{\underset{\text{fused}}{𝐃}}=\frac{\sum_{f\in F}w_{f}\cdot \overset{i}{\underset{\text{cls},f}{𝐃}}}{\sum_{f\in F}w_{f}},$$ (5)

where F={Name,Synonymous,Definitions} represents the set of textual fields, and $w_{f}$ denotes the weight assigned to the field f to reflect the relative importance of different information sources. The fused representation $\overset{i}{\underset{\text{fused}}{𝐃}}\in {\mathbb{R}}^{768}$ is further projected to a 128-dimensional space through a linear transformation to obtain the final disease embedding ${𝐃}^{i}\in {\mathbb{R}}^{128}$, providing a compact and informative representation for downstream drug-disease association prediction.

#### Attention-based pooling for molecular embeddings.

After obtaining token-level molecular representations $\overset{d}{\underset{\text{seq}}{𝐌}}\in {\mathbb{R}}^{L_{d}\times 384}$, we use an attention-weighted pooling mechanism [29] to aggregate the sequence into a fixed-size drug-level representation. The attention pooling process is calculated as:


$$e_{j}=\text{MLP}\left({𝐦}_{j}\right)=W_{\text{2}}\cdot tanh(W_{\text{1}}\cdot {𝐦}_{j}+b_{\text{1}})+b_{\text{2}},$$ (6)

$$\alpha_{j}=\frac{exp(e_{j}-{max}_{k}e_{k})}{\sum_{k=1}^{L_{d}}exp(e_{k}-{max}_{k}e_{k})},$$ (7)

$${𝐌}^{d}=\sum_{j=1}^{L_{d}}\alpha_{j}\cdot {𝐦}_{j},$$ (8)

where ${𝐦}_{j}\in {\mathbb{R}}^{384}$ represents the embedding of thej- th token from $\overset{d}{\underset{\text{seq}}{𝐌}}$, $\;e_{j}$ denotes the attention score received by a two-layer feedforward network with tanh activation, and $\alpha_{j}$ is the normalized attention weight. The subtraction of ${max}_{k}e_{k}$ in the softmax operation ensures numerical stability. The final pooled representation ${𝐌}^{d}\in {\mathbb{R}}^{384}$ aggregates information from all tokens, with each token weighted by its learned attention coefficient. This attention mechanism allows the model to automatically identify and emphasize the most informative molecular substructures for drug-disease association prediction.

#### Contrastive learning for feature enhancement.

To enhance the prediction power of drug and disease representations, we incorporate a contrastive learning framework [30] that projects embeddings into a shared latent space. Two projection networks $f_{\text{drug}}$ and $f_{\text{disease}}$ are used to transform the original embeddings:


$$\overset{d}{\underset{\text{drug}}{𝐙}}=f_{\text{drug}}\left({𝐌}^{d}\right),\text{\textbackslash{}hspace\{1em}\}\overset{i}{\underset{\text{disease}}{𝐙}}=f_{\text{disease}}\left({𝐃}^{i}\right),$$ (9)

where $\overset{d}{\underset{\text{drug}}{𝐙}}$ and $\overset{i}{\underset{\text{disease}}{𝐙}}\in {\mathbb{R}}^{128}$ represent the projected embeddings. Both projection networks consist of two linear layers with ReLU activation and layer normalization. An interaction function$f_{\text{interact}}$ operates on the concatenated projected features to capture cross-modal patterns:


$${𝐙}_{\text{interact}}=f_{\text{interact}}\left(\left[\overset{d}{\underset{\text{drug}}{𝐙}};\overset{i}{\underset{\text{disease}}{𝐙}}\right]\right),$$ (10)

The final enhanced representation combines the original and projected features:


$${𝐗}_{\text{enhanced}}=[{𝐌}^{d};{𝐃}^{i};\overset{d}{\underset{\text{drug}}{𝐙}};\overset{i}{\underset{\text{disease}}{𝐙}};{𝐙}_{\text{interact}}],$$ (11)

The projection networks are optimized using an InfoNCE-based contrastive loss. For a batch of drug-disease pairs, we compute the similarity matrix:


$$S_{ij}=\frac{\overset{i}{\underset{\text{drug}}{𝐙}}\cdot (\overset{j}{\underset{\text{disease}}{𝐙}}{)}^{T}}{\tau },$$ (12)

where τ is the temperature parameter. The contrastive loss with high similarity is used for separating positive pairs from negative ones:


$${ℒ}_{\text{contrast}}=-\sum_{\left(d,i\right):y_{di}=1}log\frac{exp\left(S_{di}\right)}{\sum_{j}exp\left(S_{dj}\right)},$$ (13)

This strategy enables learning of discriminative representations that capture both entity-specific properties and relational patterns between drugs and diseases.

#### Random forest for drug-disease association prediction.

We use Random Forest to predict potential drug-disease associations based on the PLM-derived embeddings. Random Forest is an ensemble learning algorithm that constructs multiple decision trees through bootstrap sampling and aggregates their predictions via majority voting. For each drug-disease pair with enhanced feature representation ${𝐗}_{\text{enhanced}}$, the prediction result is computed as:


$$\hat{y}=\frac{\text{1}}{T}\sum_{t=1}^{T}h_{t}\left({𝐗}_{\text{enhanced}}\right),$$ (14)

whereT is the number of trees and $h_{t}$ denotes the output of thet -th tree. Each tree is trained on a bootstrap sample with random feature subsets at each split, providing robustness against overfitting.

#### Optimal threshold selection via Youden index.

To convert probability scores into binary predictions, we apply the Youden index to determine the optimal threshold. The Youden index maximizes the sum of sensitivity and specificity as:


J(θ)=TPR(θ)−FPR(θ), (15)

$$\theta^{*}={argmax}_{\theta }J\left(\theta \right),$$ (16)

where TPR and FPR denote true positive rate and false positive rate, respectively. Binary predictions are obtained via ${\hat{y}}_{\text{binary}}=1(\hat{y}\geq \theta^{*})$.

## Results

### A. Experimental setting

In this section, known drug-disease associations are labeled as positive samples, while pairs without known association information serve as negative samples. Given the limited number of positive samples, we apply a balanced sampling strategy by randomly selecting an equal number of unknown associations as negative samples. This approach mitigates potential bias caused by data imbalance and ensures a fair evaluation of model performance. We use five-fold cross-validation (5-CV) for performance assessment. The dataset is evenly split into five subsets, with four used for training and one for testing in each fold, ensuring that every sample is used for both training and testing exactly once. This procedure is repeated five times. We calculate AUC, AUPR, F1-score and Accuracy for performance evaluation.

### B. Hyperparameter configuration

**Fig 2:**
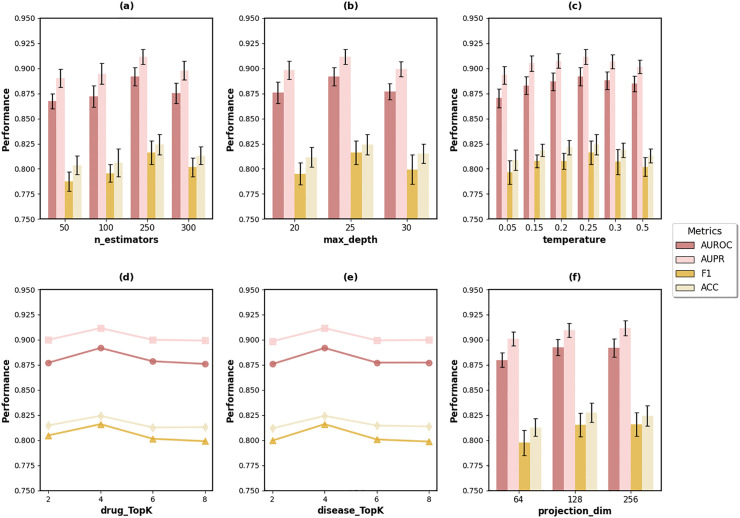
Hyperparameter sensitivity analysis of DMAPLM. Optimal configuration: n_estimators = 250, max_depth = 25, temperature = 0.25, TopK = 4, and projection_dim = 256.

We apply grid search to evaluate different hyperparameter settings on prediction performance based on 5-fold cross-validation. We conduct sensitivity analysis on key hyperparameters including n_estimators, max_depth, temperature, drug_TopK, disease_TopK, and projection_dim. The results are shown in Fig 2.

Based on the experimental results, the key hyperparameters are set as follows: The Random Forest uses 250 estimators with a maximum depth of 25. The minimum samples required to split an internal node is 5, and the minimum samples required at a leaf node is 2. Feature selection uses the square root of total features at each split. Class weights are balanced to address data imbalance. For similarity network construction, disease TopK is set to 4 and drug TopK is set to 4 to select the most relevant neighbors. The contrastive learning module uses a temperature parameter of 0.25 and projection dimension of 256. Training employs a learning rate of 0.0005 with early stopping to prevent overfitting.

### C. Ablation tests

We conduct ablation studies based on 5-fold cross-validation (Table 1) to assess the performance contribution of different components in DMAPLM. Removing pre-trained language model embeddings causes dramatic performance decline, demonstrating the critical importance of PLM representations. Replacing PLM embeddings with one-hot encoding shows moderate performance but significant degradation from PLM features. Excluding contrastive learning leads to performance reduction, confirming its importance in learning discriminative representations. Deleting attention-weighted pooling causes performance reduction, highlighting the value of adaptive feature aggregation.

**Table 1:** Results of ablation tests based on 5-fold cross-validation.

Method	AUC	AUPR	F1-score	Accuracy
**DMAPLM**	**0.8919**	**0.9116**	**0.8161**	**0.8244**
DMAPLM w/o PLM	0.5950	0.6012	0.5555	0.6220
DMAPLM w/o CL	0.8462	0.8711	0.7682	0.7877
DMAPLM w/o Att	0.8875	0.9069	0.8079	0.8144
DMAPLM w/ One-hot	0.7642	0.7644	0.7076	0.7405

DMAPLM w/o PLM is the version that removes pre-trained language model embeddings.

DMAPLM w/o CL is the version that deletes contrastive learning enhancement.

DMAPLM w/o Att is the version that removes attention-weighted pooling.

DMAPLM w/ One-hot is the version that replaces PLM embeddings with one-hot encoding.

### D. Model comparison

We further compare the prediction performance of DMAPLM with six state-of-the-art (SOTA) models using the benchmark dataset based on 5-fold cross-validation. The baseline models for performance comparison include four deep learning SOTA models and two traditional machine learning models:

- LAGCN [31]: A model that incorporates layer attention mechanisms in graph convolutional layers to enhance drug-disease association prediction.
- HGTDR [32]: A heterogeneous graph transformer model that constructs knowledge graphs and utilizes transformer networks with fully connected layers for drug repurposing prediction.
- DTI-LM [20]: A pretrained language model framework that integrates molecular graph and protein sequence features using graph attention networks for drug-target interaction prediction.
- HNF-DDA [21]: A transformer-style heterogeneous network embedding model that employs subgraph contrastive learning and all-pairs message passing for drug-disease association prediction.
- DrugLAMP [22]: A multi-modal framework combining pretrained language models with pocket-guided co-attention and paired multi-modal attention mechanisms for drug-target interaction prediction.
- Node2Vec [33]: A network embedding method that learns continuous feature representations for nodes through biased random walks.

Among these SOTA models, all focus on the broad field of drug repositioning area, with HGTDR and HNF-DDA specifically for drug-disease association prediction, while DTI-LM and DrugLAMP were designed for drug-target interaction prediction. To ensure a fair comparison, all tests are conducted under identical conditions, including five-fold cross-validation, consistent random seed initialization, and the same data partitioning strategy.

**Fig 3:**
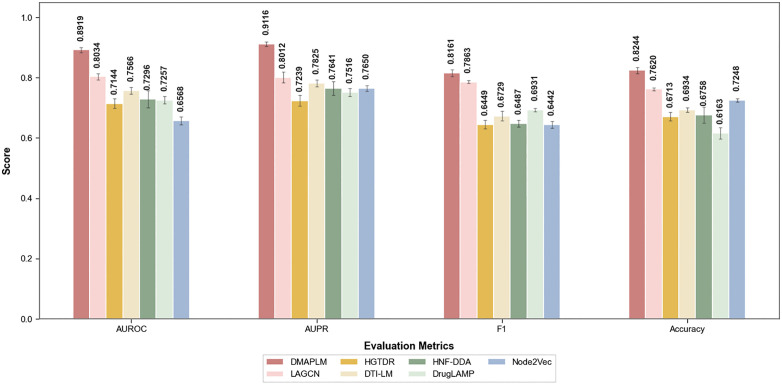
Performance comparison of DMAPLM with baseline models. DMAPLM significantly outperforms all other methods, with most notable improvements in AUROC and AUPR over the second-best model LAGCN.

As shown in Fig 3, DMAPLM outperforms all other competing models across all evaluation metrics. It achieves an AUROC of 0.8919 and AUPR of 0.9116, exceeding the second-best performing model LAGCN by 8.85% in AUROC (0.8919 vs 0.8034) and 9.22% in AUPR (0.9116 vs 0.8194). These results demonstrate that DMAPLM effectively integrates pretrained language model embeddings with contrastive learning and provides significant improvement in predicting drug-disease associations.

### E. Cold-start prediction

We conduct comprehensive cold-start prediction experiments using the standard C1, C2, and C3 protocols following established practices in computational drug discovery [34,35]. These protocols evaluate three critical scenarios that simulate real-world drug discovery challenges: C1 simulates new drug scenarios by randomly selecting 20% of drugs and their interactions as the test set; C2 evaluates new disease scenarios using 20% of diseases; C3 represents the most challenging double cold-start scenario by simultaneously selecting 20% of both drugs and diseases for testing.

**Fig 4:**
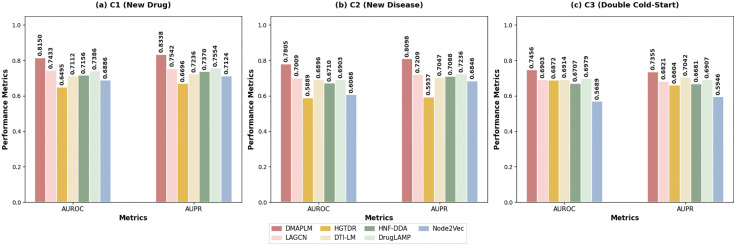
Cold-start prediction performance evaluation under three different scenarios. C1 tests new drugs, C2 tests new diseases, C3 tests both simultaneously. DMAPLM achieves best performance across all scenarios, maintaining stability in the most challenging C3.

As shown in Fig 4, DMAPLM achieves the best performance in all cold-start scenarios: AUROC and AUPR are 0.8150 and 0.8338 for C1 (new drugs), 0.7805 and 0.8098 for C2 (new diseases), and 0.7456 and 0.7355 for C3 (double cold-start). Even in the most challenging C3 scenario, DMAPLM maintains stable prediction performance, demonstrating its generalization advantage in practical drug repurposing applications.

### F. Top-K evaluation

To evaluate the prioritization ability of DMAPLM in practical drug screening, we use Top-K metrics to simulate clinical decision-making scenarios, including Precision@K (P@K), Recall@K (R@K), Mean Reciprocal Rank (MRR), and Normalized Discounted Cumulative Gain (NDCG@10).

As shown in Table 2, DMAPLM achieves superior performance on all Top-K metrics. Specifically, in the most critical Top-1 prediction, DMAPLM’s P@1 reaches 0.8139, a 3.73% improvement compared to the second-best model LAGCN (0.7846), indicating that our model can accurately identify the most promising candidate drugs. Meanwhile, the MRR value of 0.8990 (1.59% higher than LAGCN’s 0.8849) confirms the model’s advantage in early ranking, which is crucial for reducing downstream experimental validation costs. In addition, the high P@3 and R@3 scores (0.5155 and 0.9087) both surpass suboptimal methods, indicating that DMAPLM maintains high precision and recall when expanding the candidate list, making it suitable for clinical scenarios.

**Table 2:** Top-k metrics comparison.

Model	P@1	R@1	P@3	R@3	MRR	NDCG@10
**DMAPLM**	**0.8139**	**0.5770**	**0.5155**	**0.9087**	**0.8990**	**0.6626**
LAGCN	0.7846	0.5566	0.5108	0.9068	0.8849	0.6542

### G. Robustness analysis

We test the robustness of DMAPLM by systematically introducing Gaussian noise to the input features to assess the model’s resilience to data quality issues commonly encountered in biomedical fields. Gaussian noise with varying intensities (0%, 10%, 20%, 30%, 40%, 50%) is systematically injected into both the PLM embeddings and the final concatenated features during training and testing phases. The noise level represents the standard deviation of the Gaussian noise relative to the feature standard deviation.

**Fig 5:**
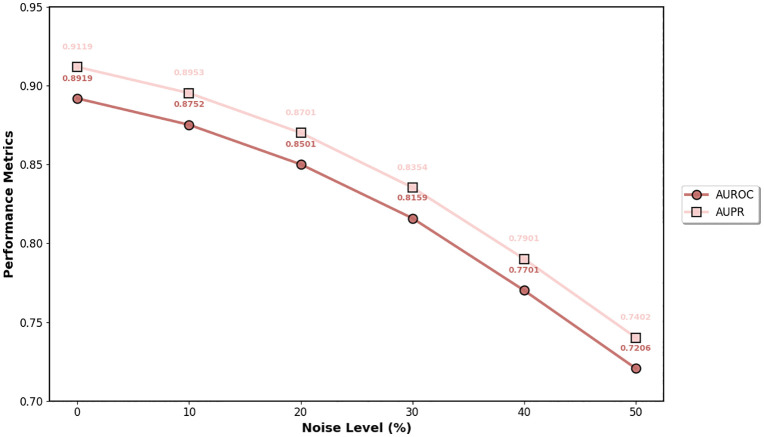
Robustness analysis under varying noise levels. Even though, results show progressive performance degradation, DMAPLM still maintains reasonable prediction ability under severe noise.

As shown in Fig 5, DMAPLM demonstrates progressive performance degradation as noise levels increase. Under moderate level (20%), the model maintains strong performance with only 4.69% AUROC degradation. Even under severe noise (50%), the model retains reasonable prediction capability with 19.21% AUROC and 18.83% AUPR degradation. The results indicate that DMAPLM’s learned representations are robust to input perturbations and can maintain clinically relevant performance when faced with noisy biomedical data.

### H. Feature quality assessment

To assess the effect of contrastive learning, we analyze similarity distributions in the embedding space. From one representative training fold, we sample 1,000 positive (known drug-disease) and 1,000 negative pairs, comparing three settings: Baseline, PLM, and PLM + CL.

Cosine similarity was used to measure feature separability [30], following the principle that positive pairs should be close while negative pairs should be distant. A single-fold analysis was used to clearly show the distributional differences without averaging effects.

**Fig 6:**
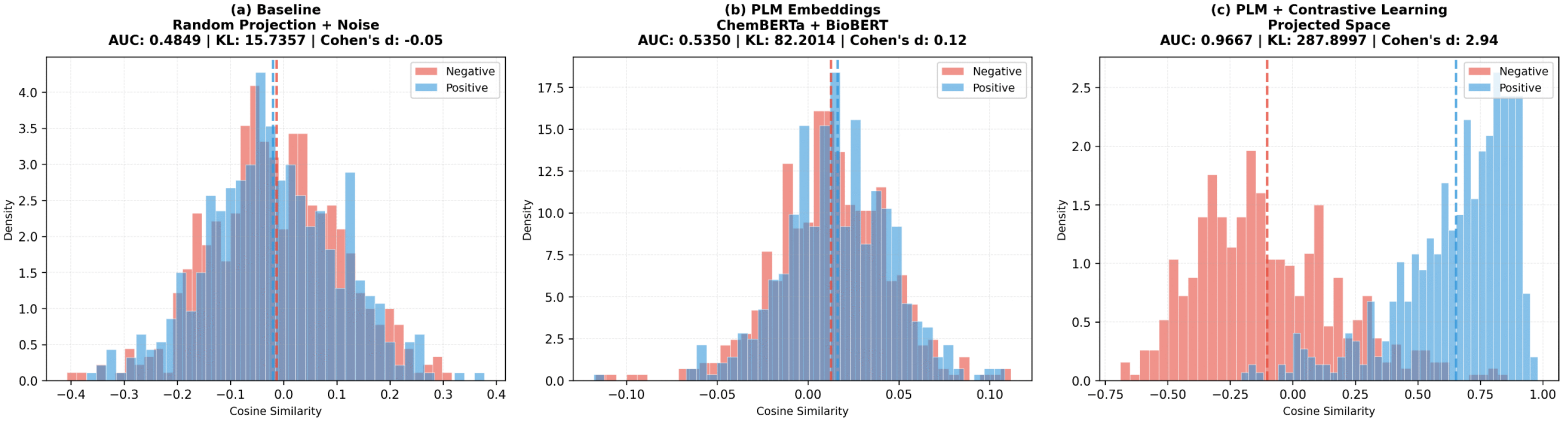
Cosine similarity distributions for feature quality assessment. Baseline shows severe overlap; PLM achieves partial separation; PLM + CL reaches clear boundaries.

As shown in Fig 6, contrastive learning significantly improves the discriminability of the embedding space. Visualization shows that Baseline embeddings exhibit strong overlap between positive and negative samples; PLM introduces partial separation, while PLM + CL achieves clear boundaries. Quantitative analysis confirms this improvement: positive pair similarity for PLM + CL reaches 0.679 (PLM: 0.346), negative pair similarity decreases to 0.146 (PLM: 0.182), and Cohen’s d attains 4.56, far exceeding PLM (2.18) and Baseline (0.24) [36]. This enhanced separability results in a cosine similarity-based classification AUC of 0.915, representing 21.5% and 62.2% improvements over PLM and Baseline, respectively, confirming that contrastive learning produces highly discriminative features that could support accurate association prediction.

### I. PLM-based embedding clustering analysis

We obtain MeSH Tree Numbers for diseases via the NLM REST API [https://id.nlm.nih.gov/mesh/] [37]. The first three characters of each Tree Number define the Primary_Class: C01 (bacterial and fungal infections), C04 (neoplasms), C10 (nervous system diseases), C14 (cardiovascular diseases), C17 (skin and connective tissue diseases), and C23 (pathological conditions and signs). Of the 873 diseases with retrieved Tree Numbers, the top 6 categories contain 600 diseases (70%): C01 (160), C04 (122), C10 (103), C23 (76), C14 (72), and C17 (67).

We perform KMeans clustering (k = 6) on disease embeddings. Table 3 shows PLM embeddings achieve NMI = 0.342, ARI = 0.367, and Silhouette = 0.174. TF-IDF obtains NMI = 0.027, ARI = -0.004, and random baseline achieves NMI = 0.011, ARI = 0.001. PLM embeddings significantly outperform baselines in capturing disease taxonomy.

**Table 3:** Disease Embedding Clustering Metrics.

Method	NMI	ARI	Silhouette
**PLM**	**0.342**	**0.367**	**0.174**
TF-IDF	0.027	-0.004	-0.183
Random	0.011	0.001	0.006

**Fig 7:**
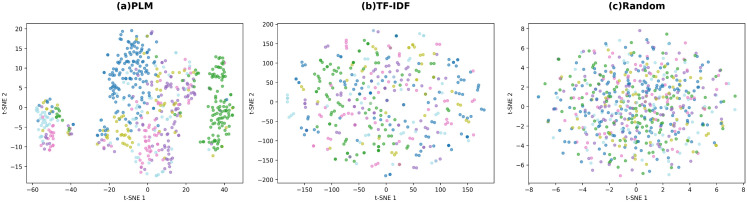
t-SNE Visualization of Disease Embeddings. PLM displays clear clustering with distinct category boundaries; TF-IDF and Random show no patterns.

Fig 7 shows t-SNE projections of embeddings. PLM embeddings display clear cluster structures with distinct category separation. TF-IDF and random embeddings show no clear patterns. The visualization confirms that PLM effectively encodes disease features consistent with MeSH taxonomy.

### J. Attention weight visualization

**Fig 8:**
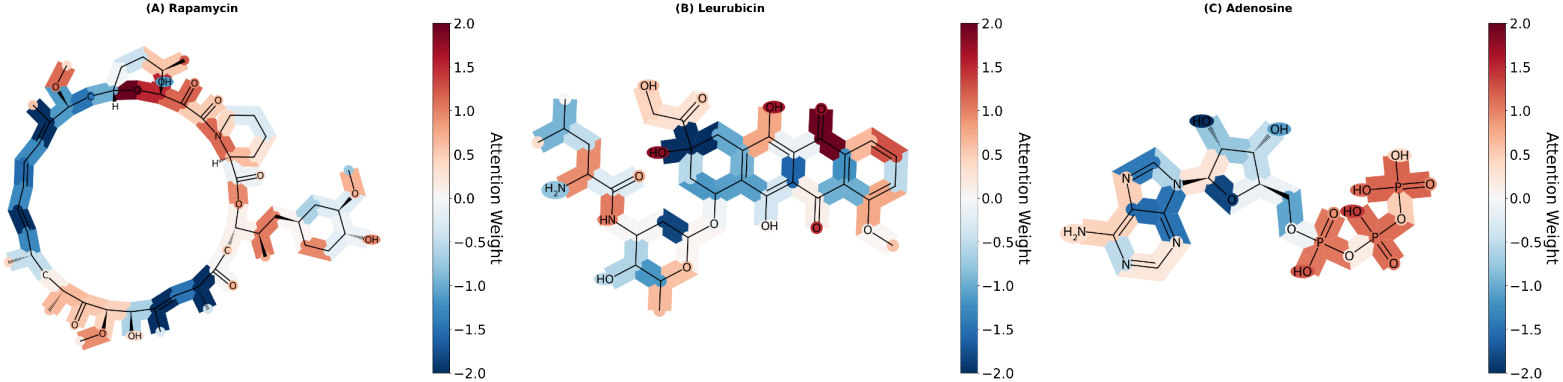
Attention weight visualization of three drugs.

We apply attention weight visualization to demonstrate the interpretability of our model. We select three drugs (Rapamycin, Leurubicin and Adenosine) as examples. Fig 8 shows that our model automatically identifies pharmacologically relevant substructures. When predicting Rapamycin’s association with prostate cancer, attention concentrates on the macrolide core and epoxide group-precisely the FKBP12-binding pharmacophore confirmed by crystal structures. Meanwhile, for Leurubicin and Adenosine against bacterial infection, attention patterns clearly differentiate their distinct mechanisms (aromatic ring systems vs. phosphate chains), indicating that DMAPLM can learn mechanistic features of drugs.

### K. Case studies

To test DMAPLM’s ability to predict novel drug-disease associations under realistic scenarios, we conduct case studies using the highest-scoring predictions obtained from the C1 and C2 cold-start validation protocols described in Section E. Specifically, the top-ranked associations involved Rapamycin-Prostate cancer, Leurubicin-Acute myelogenous leukaemia, and Adenosine-Bacterial infection. In each case, the original known associations are removed from the binary interaction matrix to simulate unknown conditions, and DMAPLM is then applied to predict new links. The resulting top predictions are validated through systematic literature mining on PubMed (https://pubmed.ncbi.nlm.nih.gov/) using PMID-indexed evidence.

As shown in Table 4, all the top five predictions for the three drugs have been confirmed by literature. For example, Shorning et al. [38] demonstrated rapamycin’s therapeutic potential in prostate cancer by highlighting the critical role of the PI3K–AKT–mTOR signaling axis in this malignancy. Breistøl et al. [39] confirmed the antitumor efficacy of N-L-leucyl-doxorubicin (Leurubicin) in human melanoma xenograft models, demonstrating superior tumor growth inhibition compared to doxorubicin. Importantly, the study verified that Leurubicin functions as a prodrug and achieves higher intratumoral concentrations of active doxorubicin, thereby enhancing therapeutic efficacy. Leurubicin is an N-L-leucyl prodrug of the anthracycline doxorubicin, exhibiting antineoplastic activity (PubChem CID: 68897). Xiang et al. [40] confirmed adenosine’s protective role against bacterial infection through NLRP3 inflammasome activation.

**Table 4:** The top five diseases predicted by DMAPLM for three drugs.

Drug	Disease	Rank	Evidence
Rapamycin	Prostate cancer	1	PMID:32630372
Rapamycin	Advanced cancer	2	PMID:22149876
Rapamycin	Lung cancer	3	PMID:37057884
Rapamycin	Plasma cell myeloma	4	PMID:39875173
Rapamycin	Intracranial meningioma	5	PMID:30082628
Leurubicin	Bacterial infection	1	PMID:37308748
Leurubicin	Acute myelogenous leukaemia	2	PMID:40293351
Leurubicin	Leukemia	3	PMID:20572037
Leurubicin	Chronic obstructive pulmonary disease	4	PMID:6254538
Leurubicin	Melanoma (Melanoma and Bacterial infection are two different diseases. In fact, Bacterial infection is the 1st prediction result of Adenosine, which means that it should be placed in the following cell in the table, while we put them together in this cell. Bacterial infection is therefore needed to be deleted here. We reported this mistake to **anitha.samidurai@editorialoffice.co.uk** on 11th April, 2026.)	5	PMID:10533461
Adenosine	Bacterial infection	1	PMID:23717478
Adenosine	MALT lymphoma	2	PMID:36539557
Adenosine	Plasma cell myeloma	3	PMID:32409420
Adenosine	Hepatosplenic T-cell lymphoma	4	PMID:2981904
Adenosine	Myeloproliferative neoplasm	5	PMID:33748423

We further test three drugs with mixed therapeutic and adverse profiles and validate their top predicted disease associations. As shown in Table 5, most of these predictions show strong literature support. For example, Hong et al. [41] demonstrated both atorvastatin and rosuvastatin’s therapeutic efficacy in coronary heart disease, showing that high-intensity statin treatment with either atorvastatin 40 mg or rosuvastatin 20 mg daily significantly reduced the 3-year composite risk of death, myocardial infarction, stroke, or coronary revascularization in a randomized trial of 4,400 patients with established coronary artery disease. Haidar et al. [42] confirmed pramlintide’s therapeutic effectiveness against type 1 diabetes, achieving increased time in glycemic range from 74% to 84% (P = 0.0014) and improved daytime control in a randomized crossover trial using a novel dual-hormone artificial pancreas system. Wald et al. [43] confirmed dexamethasone’s therapeutic effectiveness against bacterial meningitis in children, achieving improved mortality and neurologic outcomes in pneumococcal meningitis as an adjuvant therapy. We note that these computational predictions primarily reflect therapeutic associations, though rare instances may represent adverse events (e.g., hydrocortisone-associated meningitis [44]). Experimental validation is required to determine the clinical nature of each prediction.

**Table 5:** The top five drugs predicted by DMAPLM for three diseases.

Disease	Drug	Rank	Evidence
Coronary Heart Disease	Atorvastatin	1	PMID:36877807
Coronary Heart Disease	Rosuvastatin	2	PMID:36877807
Coronary Heart Disease	Enalapril	3	PMID:41070303
Coronary Heart Disease	Esmolol	4	PMID:26847114
Coronary Heart Disease	Bumetanide	5	NA
Type-1 Diabetes	Pramlintide	1	PMID:31974099
Type-1 Diabetes	Dapagliflozin	2	PMID:32311204
Type-1 Diabetes	Exenatide	3	PMID:32573927
Type-1 Diabetes	Simvastatin	4	PMID:17519305
Type-1 Diabetes	Atorvastatin	5	PMID:37951886
Meningitis	Dexamethasone	1	PMID:40874187
Meningitis	Betamethasone	2	PMID:27404370
Meningitis	Ciprofloxacin XR	3	PMID:29944651
Meningitis	Fusidic Acid	4	PMID:35810737
Meningitis	Hydrocortisone	5	PMID:26358724

To validate DMAPLM’s predictions for rapamycin’s anticancer activity, we conduct molecular docking studies. Rapamycin (also known as sirolimus), a macrocyclic lactone isolated from Easter Island soil, was initially discovered as an antifungal agent but later recognized for its potent immunosuppressive and antiproliferative properties [45,46].

Rapamycin’s mechanism of action is well-established: it binds the intracellular receptor FKBP12, and this complex then binds the FRB (FKBP12-Rapamycin Binding) domain of mTOR, allosterically inhibiting mTOR kinase activity [47]. In 1996, Choi et al. solved the crystal structure of the FKBP12-rapamycin-FRB ternary complex (PDB: 1FAP) [48], followed by Liang et al.’s refined 2.2 Å resolution structure (PDB: 4FAP) in 1999 [49].

The FDA approved rapamycin (1999) and its analogs temsirolimus (2007) and everolimus (2009) as mTOR inhibitors for organ transplantation and multiple cancers [50,51]. Moreover, dysregulation of the mTOR signaling pathway has been widely observed across diverse human cancers [52,53], particularly in prostate cancer, lung cancer, and plasma cell myeloma-the cancer types predicted by DMAPLM for rapamycin treatment.

We use the high-resolution FKBP12-rapamycin-FRB ternary complex structure (PDB: 4FAP) as the docking template. The protein structure is prepared using AutoDockTools 1.5.6, including addition of polar hydrogens, Gasteiger charge calculation, and conversion to PDBQT format. Rapamycin’s 3D structure is obtained from PubChem (CID: 5284616) and processed with Open Babel for energy minimization.

Molecular docking is performed using AutoDock Vina v1.2.7. The search space includes the entire FRB domain and surrounding regions, with exhaustiveness set to 8 for thorough conformational sampling. The software generates 9 binding conformations ranked by binding affinity. Results are visualized and analyzed using PyMOL 3.1.

AutoDock Vina generates 9 rapamycin-mTOR binding conformations (Table 6). Binding affinities ranged from -6.807 to -9.921 kcal/mol. The best conformation (Mode 1) shows a binding affinity of -9.921 kcal/mol, indicating strong binding capability. The top five conformations all exceed -7.5 kcal/mol with RMSD values <2.5 Å, demonstrating consistent binding modes. This binding energy falls within the moderate-to-strong binding range (-9.0 to -12.0 kcal/mol) [54], suggesting favorable bioactivity potential.

**Table 6:** Molecular docking results of Rapamycin with mTOR (PDB: 4FAP).

Model	Binding Affinity (kcal/mol)	RMSD l.b. (Å)	RMSD u.b. (Å)
1	**–9.92**	0.000	0.000
2	–8.47	2.37	6.95
3	–7.99	1.90	3.75
4	–7.50	1.80	3.58
5	–7.04	8.59	12.60

Note: RMSD l.b. and RMSD u.b. represent lower-bound and upper-bound root-mean-square deviations relative to the best conformation.

**Fig 9:**
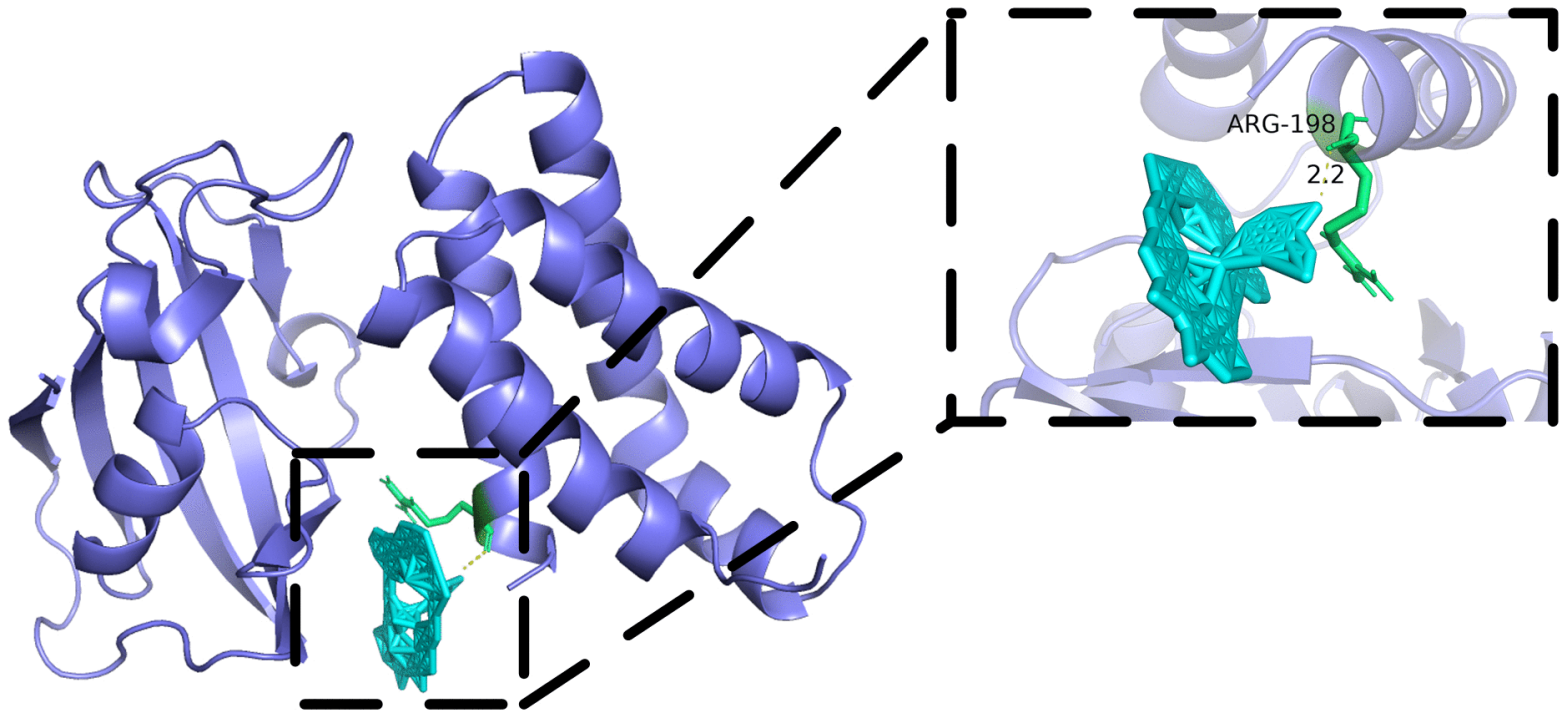
Docking pose of Rapamycin bound to the FRB domain of mTOR (PDB: 4FAP). Rapamycin docks into FRB domain, forming hydrogen bond with ARG-198 with binding affinity of -9.921 kcal/mol, validating biological plausibility of predictions.

PyMOL visualization (Fig 9) reveals that rapamycin successfully docked into the FRB domain binding pocket. Analysis identifies a key hydrogen bond between rapamycin and ARG-198 (Chain B) with a distance of 2.2 Å (Fig 9), within the ideal range for strong hydrogen bonding (2.0-3.0 Å) [55]. Additionally, rapamycin forms extensive hydrophobic interactions with the α1 and α4 helices of the FRB domain, consistent with the interaction patterns observed in Liang et al.‘s crystal structure [49].

This case study provides structural evidence supporting DMAPLM’s prediction of rapamycin’s anticancer potential. The docking results confirm strong rapamycin-mTOR interactions, consistent with its known inhibitory mechanism. Together with literature evidence on mTOR dysregulation in prostate, lung, and myeloma cancers, these findings demonstrate biological plausibility for the predicted associations.

## Discussion

In this study, we introduce DMAPLM, a multimodal pretrained framework designed to address key challenges in computational drug repositioning, including data sparsity, modality heterogeneity, and limited generalizability. By combining ChemBERTa-2–based molecular encoding with BioBERT-based disease text representation, DMAPLM enables a unified cross-modal embedding space optimized through contrastive alignment. This design allows the model to capture complementary semantic and structural information that are often overlooked by graph-based or matrix-completion approaches.

Across five-fold and cold-start evaluations, DMAPLM consistently outperformed state-of-the-art baselines, highlighting its robustness in realistic scenarios where novel drugs or diseases lack sufficient annotation. Notably, the model maintained strong performance even in the double cold-start setting, underscoring its ability to predict unobserved interaction patterns. This property is of practical relevance for repurposing tasks, where candidate compounds or emerging disease phenotypes frequently lie outside the training dataset.

The case studies further demonstrate DMAPLM’s capacity to generate biologically plausible hypotheses. High-confidence associations were subsequently supported through literature mining and molecular docking. The structural consistency between predicted binding modes and known biochemical mechanisms underscore the value of integrating pretrained language models for representing both molecular substructures and disease semantics.

A key advantage of DMAPLM lies in its interpretability. Ablation studies reveal that DMAPLM’s performance is primarily driven by pretrained semantic representations capturing both chemical and biomedical knowledge. Additionally, contrastive learning produces a discriminative embedding space where positive and negative drug-disease pairs are clearly separated, enabling interpretable similarity-based classification. Attention weight visualization of drugs further demonstrates the interpretability of our model.

Despite these strengths, several limitations remain. First, the model’s performance is inherently constrained by the availability and coverage of curated drug–disease data; missing annotations or inconsistent disease terminology may introduce bias. Second, although contrastive pretraining improves representation alignment, the resulting embeddings still reflect static molecular and textual information and do not fully capture context-dependent biological processes. Third, the validation strategy relies on *in silico* docking and literature evidence; experimental assays are required before clinical translatability can be established.

Future work may focus on incorporating dynamic biological data such as transcriptomic perturbation profiles or patient-specific multi-omics features, which may enhance interpretability and mechanistic insight. Integrating causal reasoning or pathway-aware modeling could further improve the identification of therapeutically actionable relationships. In addition, extending DMAPLM to support few-shot or zero-shot prediction paradigms may broaden its utility for understudied diseases.

## Conclusions

DMAPLM provides an effective and interpretable multimodal framework for computational drug repositioning. By leveraging pretrained language models and contrastive representation alignment, the method achieves substantial improvements in predictive accuracy and generalization across multiple evaluation settings. Case studies supported by molecular docking and literature evidence demonstrate that DMAPLM can generate biologically meaningful hypotheses with translational potential. Overall, our study offers a scalable and robust computational tool for accelerating drug repurposing, and its integration with experimental validation pipelines holds promise for facilitating more efficient therapeutic discovery.
